# The efficacy of anakinra compared to standard care of treatment for COVID-19: a meta-analysis

**DOI:** 10.55730/1300-0144.5345

**Published:** 2022-01-25

**Authors:** Hamna MALIK, Hira BINT ABDUL JABBAR, Farah LATIF, Azza SARFRAZ, Zouina SARFRAZ, Muzna SARFRAZ

**Affiliations:** 1 Research, Services Institute of Medical Sciences, Lahore, Pakistan; 2 Research, King Edward Medical University, Lahore, Pakistan; 3 Research and Publications, Fatima Jinnah Medical University, Lahore, Pakistan; 4 Pediatrics and Child Health, The Aga Khan University, Karachi, Pakistan

**Keywords:** Anakinra, cytokine storm, COVID-19, mortality, meta-analysis

## Abstract

**Background/aim:**

As SARS-CoV-2 continues to spread worldwide, this study brings to light the link that anakinra, a recombinant IL-1 receptor antagonist, has in averting grave clinical outcomes. The objectives of this meta-analysis are to investigate the effects of anakinra in interventional groups compared to control/standard of care groups on mortality along with the provision of a prevalence estimate of the variables associated with death (C-reactive protein-CRP, ferritin, acute respiratory distress syndrome-ARDS).

**Materials and methods:**

According to the PRISMA 2020 statement guidelines, a systematic search was conducted from December 19, 2020, until December 10, 2021, with keywords including COVID-19, coronavirus, SARS-CoV-2, anakinra, mortality, across the following databases: PubMed/MEDLINE, Scopus, Web of Science, CINAHL Plus, and Cochrane. A random-effects model was applied using RevMan 5.4 for all statistical analyses.

**Results:**

The meta-analysis pooled in 1297 participants with 565 (43.6%) patients in the anakinra group. When comparing to the control/standard of care group, the anakinra group had a much lower risk of death (RR = 0.47. 95% CI = 0.37–0.59, Z = 6.44; P < 0.001). In addition to the risk of death being reduced by around 50% in the interventional group, prognostic indicators such as CRP and ferritin were improved with fewer occurrences of severe ARDS.

**Conclusion:**

Patients with COVID-19 pneumonia may be treated with anakinra as a safe and viable treatment modality to defer adverse outcomes such as a death in the 28-day period. Despite an auspicious premise, our findings must be used with caution as adequately powered randomized, placebo-controlled trials are required to corroborate these findings.

## 1. Introduction

In December 2019, the severe acute respiratory coronavirus 2 (SARS-CoV-2) was identified in Wuhan, China that led to a pandemic in early 2020. Coronavirus disease 2019 (COVID-19) continues to spread across the world [1]. With the recent omicron and delta variant amid other strains emerging across countries worldwide, the clinical spectrum of COVID-19 ranges from no clinical symptoms to sepsis, organ dysfunction, and ultimately death [2]. While the presence of the many available vaccines has offered certain protective benefits, effective antiinflammatory treatment is required as a viable treatment modality to ensure that mortality rates are decreased [3].

Inflammation plays a pivotal role in the pathophysiology of COVID-19 [4]. In line with initial reports, critical COVID-19 patients show higher proinflammatory cytokines [5]. In patients with severe hyper-inflammation, the proinflammatory cytokine interleukin-1β plays a central role in predisposing them to macrophage activation syndrome [3,5]. The activation of the endothelium may lead to hypotension, fluid extravasation, and possibly death [3]. As a recombinant IL-1 receptor antagonist (IL-1Ra), anakinra is currently a preferred treatment approach for patients with rheumatoid arthritis, Still’s disease, and cryopyrin-associated periodic syndrome [5]. With the increasing interest in anakinra as an adjuvant antiinflammatory treatment modality in COVID-19, primary clinical studies suggest that there is a decrease in mortality risk in addition to improved respiratory outcomes [2].

The objective of this meta-analysis is to investigate the effects of anakinra in interventional as compared to control/standard of care (SoC) groups on mortality, along with providing a prevalence estimate of the variables associated with death (CRP, Ferritin, ARDS).

## 2. Materials and methods

**Figure 1:**
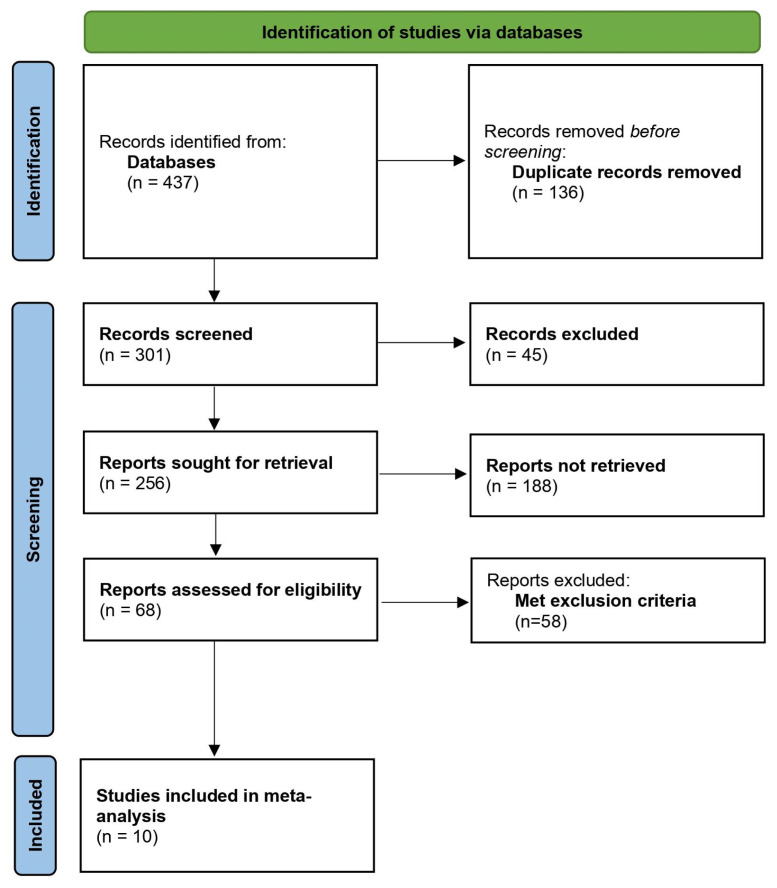
PRISMA flowchart.

As per the Preferred Reporting Items for Systematic Reviews and Meta-Analyses (PRISMA) 2020 statement [6], a systematic search was conducted from December 2019 until December 10, 2021, using keywords including COVID-19, coronavirus, SARS-CoV-2, Anakinra, and mortality using the BOOLEAN logic (and/or). The following databases were searched: PubMed/MEDLINE, Scopus, Web of Science, CINAHL Plus, and Cochrane. Preprints were omitted due to the unreliability of methodologies and the quality of the studies. The inclusion criteria comprised only quantitative primary research studies, i.e. only cohort studies, interventional studies, and clinical trials employing an interventional and a control/SoC group. Case series, case reports, letters, systematic reviews, and meta-analyses were omitted. During the systematic search, the data was entered to EndNote X9, which is a management software tool (Clarivate Analytics). An umbrella methodology was utilized where the reference lists of all screened articles were reviewed to find any studies that met the inclusion criteria. The PRISMA flowchart is depicted in Figure 1.

All investigators screened the titles and abstracts before reaching a full-text reviewal phase to determine the included studies. Any disagreements were resolved by active discussion among the investigators. An a-priori methodology was employed using a Delphi process to prioritize the outcome of interest and to collate the findings [7].

The quality appraisal for the included studies was conducted using the Grading of Recommendations, Assessment, Development, and Evaluation (GRADE) criteria. Using the tool, we evaluated the risk of biases associated with the participant selection, confounding variables, and the outcome assessment. Of all the ten studies included in this meta-analysis, there was moderate bias present due to nonrandomized and cohort study types included. We additionally checked for publication bias by generating a funnel plot.

The primary outcome included mortality from any cause in the 28 days among all studies. Since a study level outcome data for factors associated with mortality were unavailable, we provided a brief qualitative overview of the variables related to mortality and also made implications for clinical trials.

All investigators assessed the eligibility based on the inclusion criteria. The data of all studies was extracted as the author, year, study type, country, sample size, route of drug administration, and dosage of anakinra into a shared spreadsheet. For the quantitative analysis, the dichotomous data for death was enlisted into the software. This dichotomous data was presented as a risk ratio employing 95% confidence intervals. The meta-analysis was conducted using Review Manager version 5.4 (Cochrane). The findings were presented along with the I^2^ index value and the p-value (≤0.05) to test for statistically significant associations.

When computing the severity of acute respiratory distress syndrome, the Berlin modification of the American European Consensus Committee (AECC) definition was utilized with classifications as follows:

1. Mild-moderate: PaO_2_/FiO_2_ ≥ 100
2. Severe: PaO_2_/FiO_2_ ≤100

No funding was obtained for this study. Ethical approval was not obtained as only secondary clinical data was utilized for the purposes of this meta-analysis.

## 3. Results

Of the 437 studies identified across all databases, 136 duplicates were removed. Of these, 301 were screened, and 256 were sought for retrieval. With consensus among all investigators, only 69 studies were assessed for eligibility, of which 58 did not meet the inclusion criteria. In total, 9 observational studies and 1 randomized controlled trial were included in this meta-analysis (Figure 1).

The characteristics of included studies are listed in Table. Out of all studies, 3 were conducted in France and Italy respectively, two in Italy, and one each in Netherlands and Greece. Five studies employed the intravenous route of drug administration, whereas five utilized a primary subcutaneous approach (Table).

**Figure 2:**
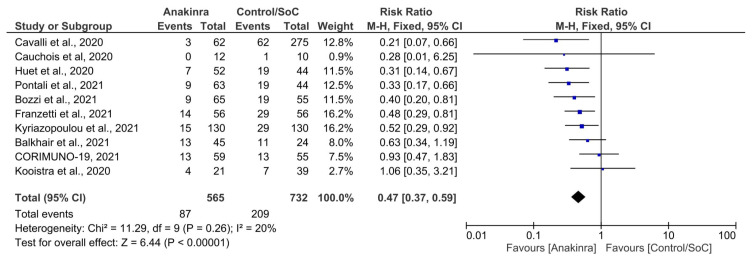
Forest plot for mortality as the primary endpoint across all included studies (N = 9).

All ten studies reported data on mortality within the 28-day period and were eligible to be included in this meta-analysis. As compared to the standard of care group, the anakinra group had a much lower risk of death (RR = 0.47. 95% CI = 0.37–0.59, Z = 6.44, P < 0.001). Using the random-effects model, the I^2^ value was 20% suggesting low levels of heterogeneity in the included studies (Figure 2). A sensitivity analysis was conducted by removing Kyriazopoulou et al., 2021 (weight = 16.2%) and Cavalli et al., 2020 (weight = 12.8%) from the funnel plot. The results were recomputed (RR = 0.5, 95% CI = 0.39–0.64, Z = 5.36, P < 0.001). The sensitivity analysis results led to similar findings as with the original analysis, where the anakinra group had a much-reduced risk of death of 50%, as compared to the SoC group, with strong statistical association (P < 0.001) (Figure 2).

On observing the factors associated with mortality in the 28 days, it was noted that 22 (11%) patients out of 208 had CRP > 100 mg/dl in the anakinra group. On comparing the results in the SoC groups, 107 (29%) of 364 patients had CRP > 100 mg/dL. These results provided insight into the lessened risk of high CRP in patients treated with anakinra as compared to standard of care groups. On noting hyperferritinemia by comparing Ferritin levels, it was found that 15 (11%) of 137 patients had > 1000 ng/mL levels in the anakinra group as compared to 31 (31%) of the 101 patients in the SoC group, suggesting that intervention reduced risks of hyperferritinemia.

The prevalence of severe ARDS (PaO_2_/FiO_2_ ≤ 100) in the anakinra group was 18 (17%) of 109 patients as compared to 49 (44%) of 95 patients in the SoC group. The results indicated that the SoC group had a higher risk of severe ARDS as compared to the anakinra group. The risk of mild to moderate ARDS was also determined (PaO_2_/FiO_2_ ≥ 200–300). It was found that the anakinra group had a 9% risk of mild-moderate ARDS (n = 19/217) as compared to a 21% risk in the SoC group (n = 72/350).

**Figure 3:**
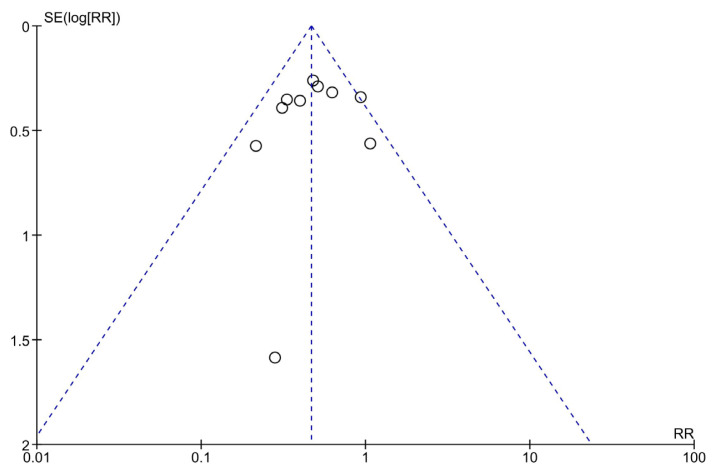
Funnel plot to visualize publication biases.

On visually inspecting the funnel plot, it may be stated that the demarcations of the 10 included studies tend to fit the criteria for an inverted funnel shape on the top, with one deviation (Cauchois et al., 2020) from the symmetrical shape. Based on these findings, it may be inferred that there was minimal publication bias present in this meta-analysis (Figure 3).

## 4. Discussion

This meta-analysis finds that the risk of death decreases by 47% for patients admitted with COVID-19 induced pneumonia to hospitals when treated with anakinra as compared to control/SoC. A randomized controlled trial addresses the efficacy of anakinra in the patient population [14]. Studies identify that the therapeutic intervention for patients with severe COVID-19 pneumonia may be confined to a therapeutic window before advanced respiratory failure occurs. The use of anakinra across several case series, cohorts, and trials has been encouraging, though not concrete for clinical use. Notably, the inflammatory biomarker thresholds used to define hyper inflammation are not uniformly reported across the observational studies included in this study. It may be stated that the identification of these biomarkers and their thresholds in advanced inflammation, or with early inflammation, may aid in administering a low or high dose of anakinra. A study conducted among those with Arab ethnicity found significant differences in mortality on the use of anakinra (24% in the intervention vs. 67% in the control group, P = 0.013). The only RCT included in this meta-analysis was conducted in France, suggesting that the availability of racial and ethnically stratified data is scarce when it comes to the assessment of anakinra as a viable treatment option for COVID-19 [18].

The CORIMUNO trial found that the requirement for invasive mechanical ventilation was reduced among those treatments with anakinra (Odds ratio = 0.38, P = 0.02), and the mortality risk was also reduced in nonintubated patients (Odds ratio = 0.32, P < 0.001) [18]. The trial’s objectives were to assess the safety and efficacy of anakinra in nonhospitalized intubated patients. To understand the correct timing of anakinra administration and adjuvant use of invasive mechanical ventilation in hospitalized intubated patients, further testing ought to be done in randomized placebo-controlled trials [18]. This will aid both low- and middle-income countries and high-income countries in overcoming the limitations of intensive care unit admissions and the limited oxygenation supplies by limiting adverse events if commenced with Anakinra before invasive mechanical ventilation is required.

A cohort study identified that patients treated with a combination of methylprednisolone and anakinra (13.9%) had lower mortality rates as compared to controls (35.6%) (P = 0.004) [11]. It may be stated that a combination treatment of steroids and anakinra may be a valid therapeutic option in COVID-19 patients presenting with respiratory failure and hyper inflammation and also in mechanically ventilated patients. Randomized controlled trials with arms for anti-IL-1 therapy and steroids alone are required to corroborate these findings.

As identified earlier, some patients with COVID-19 are likely to develop the hyperinflammatory presentation known as the cytokine storm [3]. This condition is life-threatening, requiring intensive care unit admission along with monitoring of inflammatory patterns, ferritin levels, multi-organ failure, and hemodynamic stability [2]. The primal trigger for the cytokine storm syndrome may be IL-1α. These mediators lead to an uncontrolled immune response, resulting in the production of proinflammatory interleukins [2,4]. Those who do not present with life-threatening inflammatory responses may also benefit from immunomodulating treatment options [18].

Certain limitations must be stated. This meta-analysis primarily employed observational cohort studies, with only one RCT, which raises concerns about potential confounding variables in the interventional and control groups. In the control group, the number of patients with high CRP and ferritin and the number of patients with severe lung involvement was found to be higher than the anakinra group. This may make anakinra seem like a better and more viable option than standard therapy. Various implications for clinical trials must be noted when conducting such analyses in the wake of the ongoing COVID-19 pandemic. These include establishing necessary biomarkers for evaluation pre and postintervention. Furthermore, the trials’ setting must be broadened in that groups from all racial and ethnic backgrounds be enrolled. The primary and secondary outcomes must be stratified based on the early and late use of anakinra dependent upon days from onset of symptoms and severity, the dosage and use of medication, and the invasive ventilation parameters before anakinra.

In patients with COVID-19, anakinra is a safe and viable therapeutic adjuvant to defer adverse outcomes such as death within 28 days. This meta-analysis provides moderate-quality evidence that an improvement in inflammatory markers, i.e. CRP and Ferritin lab components, are present with intervention, along with less prevalence of ARDS in the treatment groups. Our findings must be used with caution as adequately powered randomized, placebo-controlled trials are required to corroborate these findings.

## Figures and Tables

**Table:** Characteristics of included studies.

Author, year	Study type	Country	Sample size (Anakinra vs. Control/SoC)	Route of administration	Anakinra dosage
Cauchois et al, 2020 [8]	Observational	France	12 vs. 10	Intravenous	300 mg/day for 5 day, then tapered with lower dosing over 3 days
Huet et al., 2020 [9]	Observational	France	52 vs. 44	Subcutaneous	100 mg twice a day for 72 h, then 100 mg daily for 7 days
Cavalli et al., 2020 [10]	Observational	Italy	62 vs. 275	Intravenous and subcutaneous	Either 5 mg/kg twice a day IV [high dose] or 100 mg twice a day SC [low dose]
Bozzi et al., 2021[11]	Observational	Italy	65 vs. 55	Subcutaneous*	100 mg twice a day for 72 h, then 100 mg daily for 7 days SC or 5 mg/kg twice a day intravenously
Kooistra et al., 2020 [12]	Observational	Netherlands	21 vs. 39	Intravenous	300 mg anakinra intravenously, followed by 100 mg every six h
Balkhair et al., 2021 [13]	Observational	Oman	45 vs. 24	Subcutaneous	100 mg twice daily for 3 days, followed by 100 mg daily for 7 days
CORIMUNO-19, 2021 [14]	Randomized controlled trial	France	59 vs. 55	Intravenous	200 mg twice a day on days 1–3, 100 mg twice on day 4, 100 mg once on day 5
Kyriazopoulou et al., 2021[15]	Observational	Greece	130 vs. 130	Subcutaneous	100 mg once daily for 10 days
Pontali et al., 2021[16]	Observational	Italy	63 vs. 44	Intravenous	100 mg every 8 h for 3 days, with tapering
Franzetti et al., 2021 [17]	Observational	Italy	56 vs. 56	Subcutaneous*	7 d at 100 mg four times a day SC or 200 mg three times daily IV

* Intravenous approach used if the patient is on invasive mechanical ventilation
